# Supplementary material for: Single-molecule sensing of peptides and nucleic acids by engineered aerolysin nanopores
Source: Nat Commun. 2019 Oct 29;10:4918. doi: 10.1038/s41467-019-12690-9 (PMC6820719; doi:10.1038/s41467-019-12690-9)
Supplement: Supplementary file 1 — Supplementary Information [file 41467_2019_12690_MOESM1_ESM.pdf]

Supporting information for

Single-molecule sensing of peptides and nucleic acids by engineered aerolysin nanopores

Chan Cao, Nuria Cirauqui, Maria Jose Marcaida, Buglakova Elena, Alice Duperrex, Aleksandra Radenovic, Matteo Dal Peraro

Content

- Supplementary Tables 1-2
- Supplementary Figures 1-18
- Supplementary References

	WT	Ala	Trp
R282	12.3 Å	16.4 Å	14.8 Å
R220	7.8 Å	15.6 Å	9.3 Å
K238	9.8 Å	12.1 Å	11.2 Å
K242	9.4 Å	12.7 Å	11.6 Å

Supplementary Table 1 | Diameter at the R282, R220, K238 and K242 sites when these amino acids were replaced by Alanine or Tryptophan, respectively.

	EYQ3		dA_4	
	I_{res}/I_0	Width	I_{res}/I_0	Width
WT	0.500	0.040	0.450	0.005
K238A	0.550	0.033	0.520	0.009
K238Q	0.510	0.032	0.500	0.003
K238N	0.570	0.040	0.540	0.017
K238R	0.410	0.086	0.430	0.005

Supplementary Table 2 | I_{res}/I_0 and the width of half peak extracted by Gaussian fitting as EYQ3 peptide and dA_4 translocate across the wt, K238A, K238Q, K238N and K238R pores.

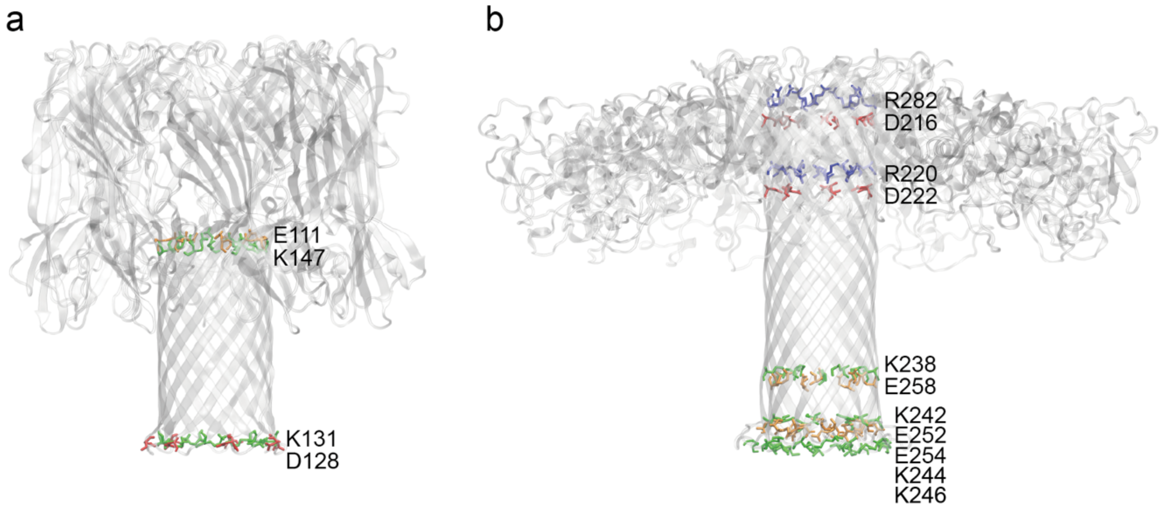

Supplementary Figure 1 | Comparison of charged amino acids lining the lumen of the α-HL (a) and aerolysin (b) pores.

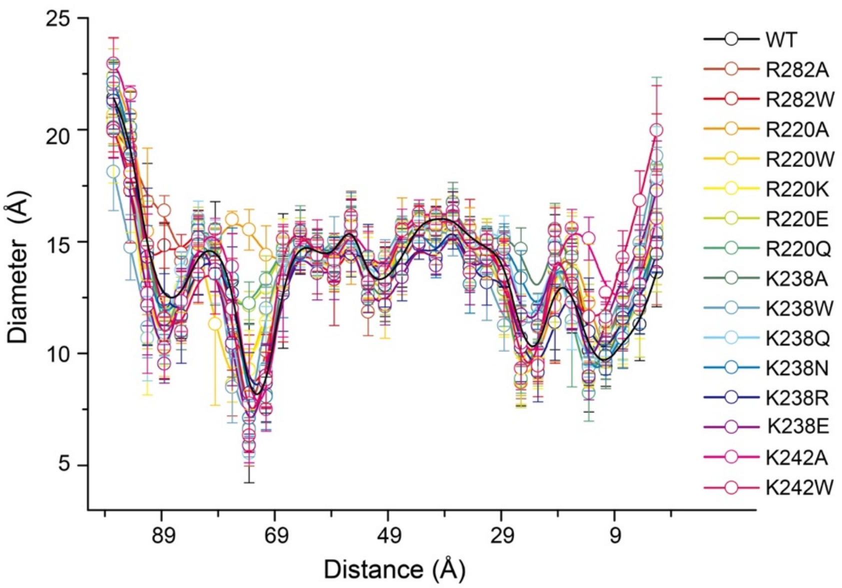

Supplementary Figure 2 | Diameter of various engineered aerolysin nanopores. The data were calculated by the PoreWalker server[1] in the direction stem to cap. Therefore, the distance = 88.5 Å represents the R282 region in the cap domain of the pore while the distance = 73.5 Å stands for the R220 region. Similarly, 22.5 Å and 10.5 Å stand for the K238 and K242 regions at the stem domain, respectively.

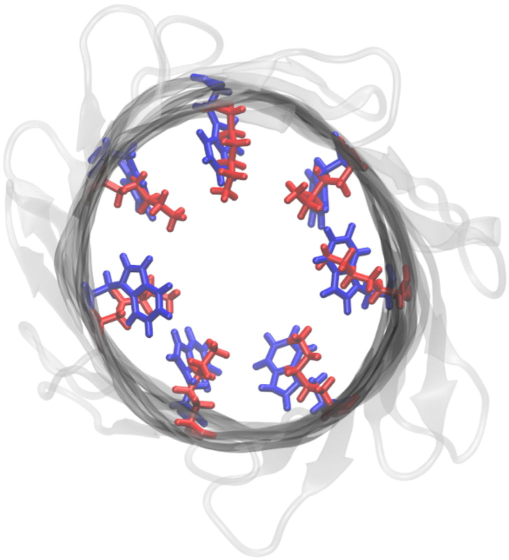

Supplementary Figure 3 | Conformation of the side chains of lysine (red) and tryptophan (blue) residues during MD simulations.

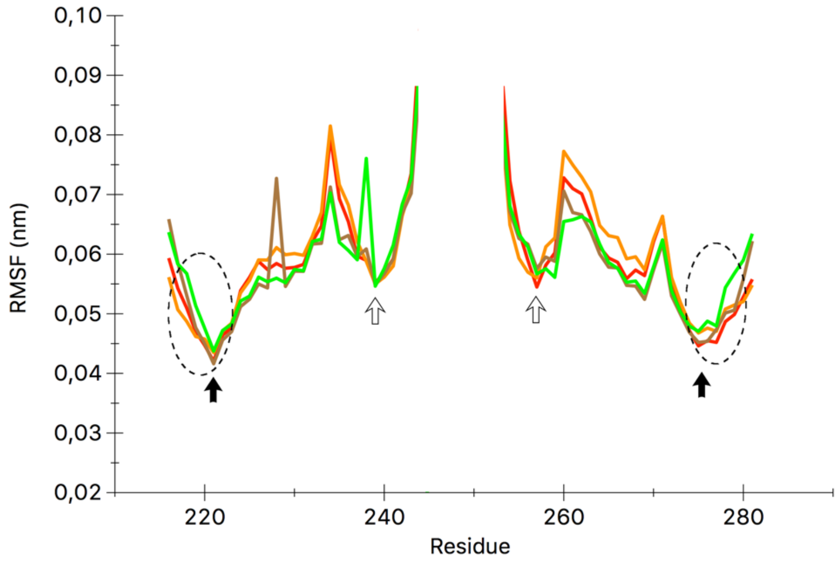

Supplementary Figure 4 | Root Mean Square Fluctuations (RMSF) of the backbone atoms of the inner barrel residues (216-244, 253-281) during the equilibrated phase of the MD simulation (10-200 ns), wt (green), K238A (red), K238Q (brown) and K238N (orange). The calculation was performed taking as a reference, for each mutant, the conformation closest to the average of the equilibrated phase. The first constriction region (around R220) is indicated with a black arrow, while the second constriction region (around K238) is indicated with a white one. The decreased fluctuations (and therefore flexibility) at the first constriction regions for K238 mutants is marked with a dashed ellipse.

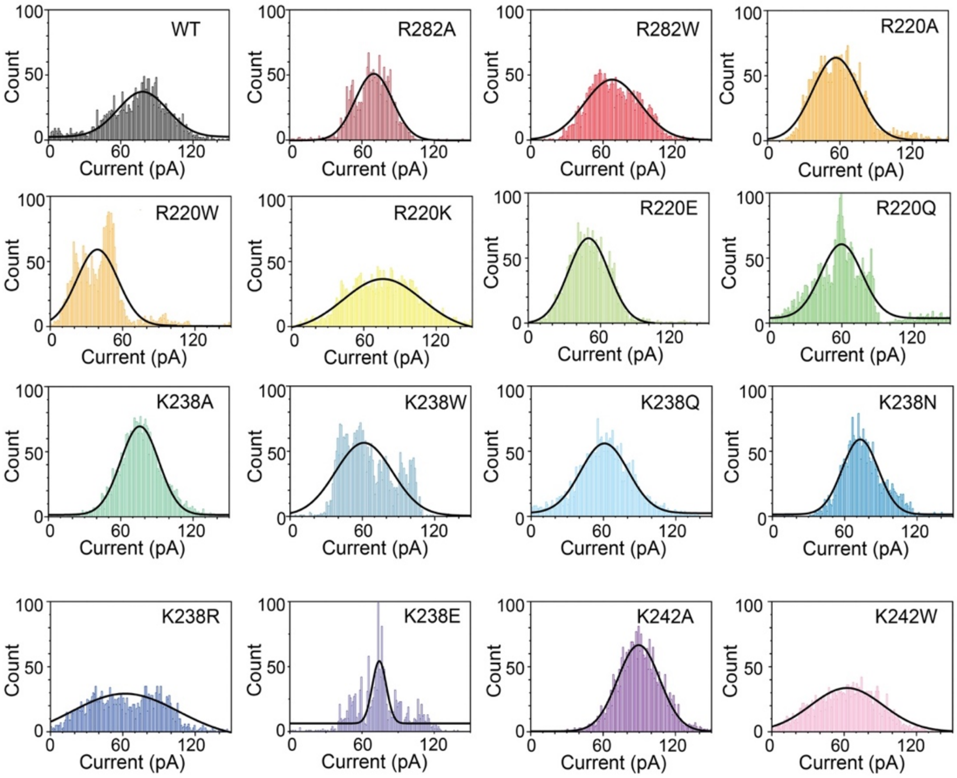

Supplementary Figure 5 | Open pore current of WT and engineered aerolysin pores at 150mV. The histograms plotted were taken over a 200 ns MD trajectory with a sampling frequency of 10 ps. The adjacent-averaging (points of window is 500) was used to smooth the data by using the ORIGIN software as we did in a previous study[2].

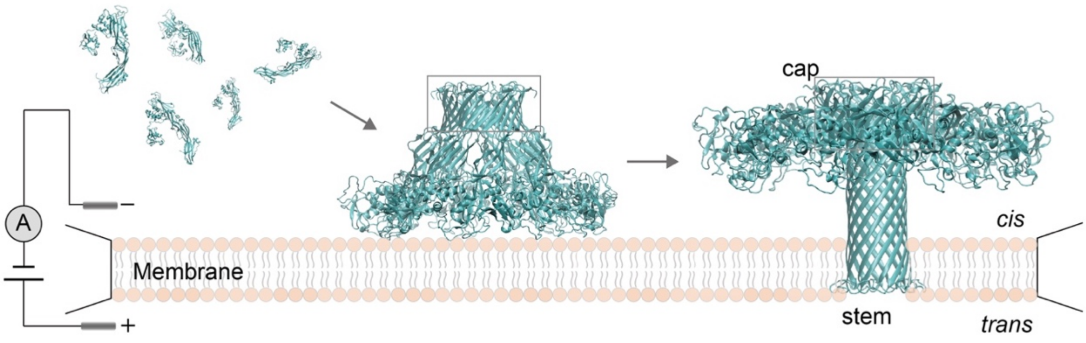

Supplementary Figure 6 | Illustration of the pore-forming process of reconstituting an engineered pore into the membrane, showing heptameric assembly of a prepore after pro-toxin activation by furins, membrane association followed by insertion of a ~10 nm long transmembrane β-barrel. Aerolysin reconstitutes in the membrane with the same orientation each time because of the large cap domain[3].

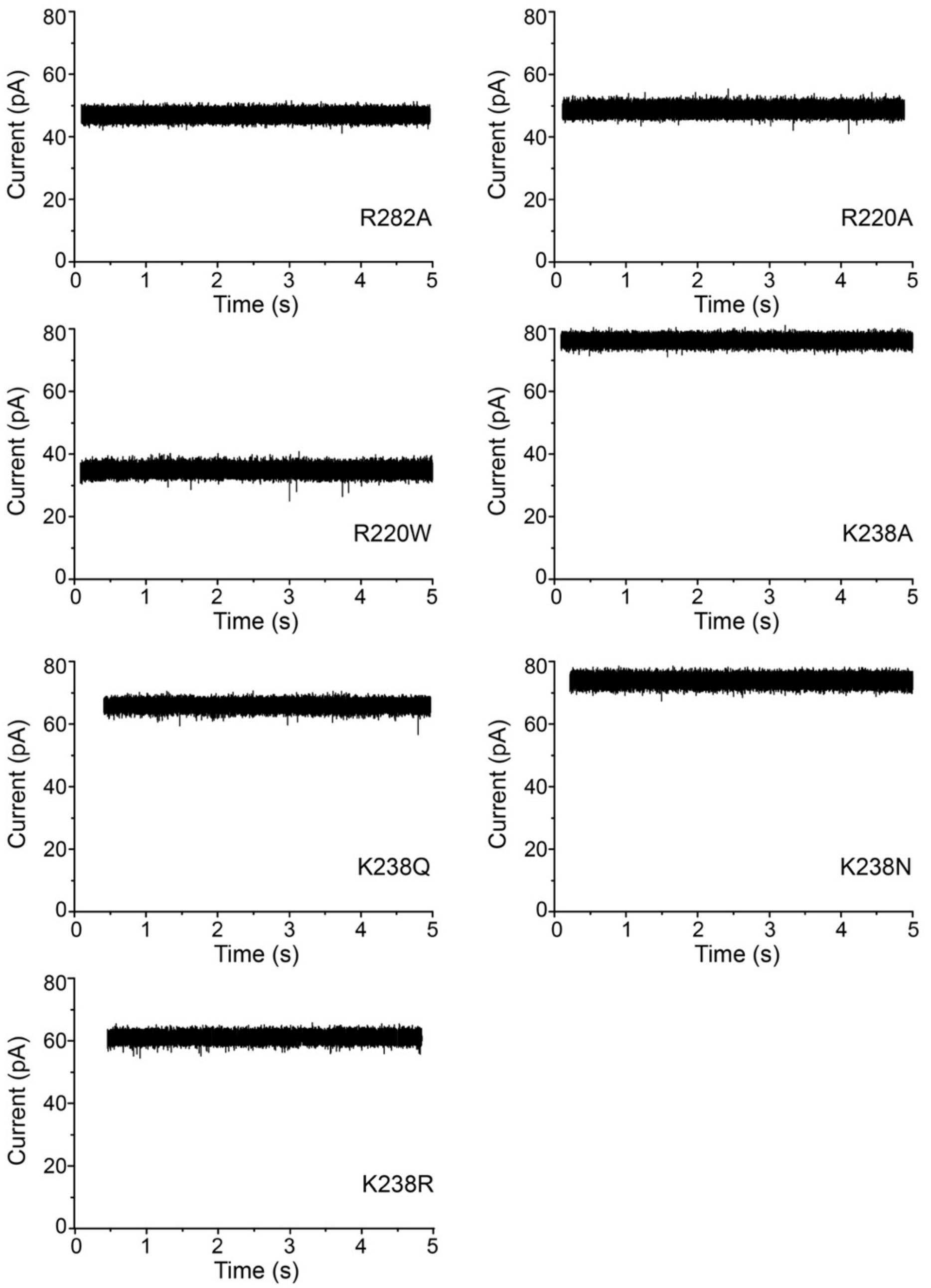

Supplementary Figure 7 | Raw current traces for individual engineered pores.

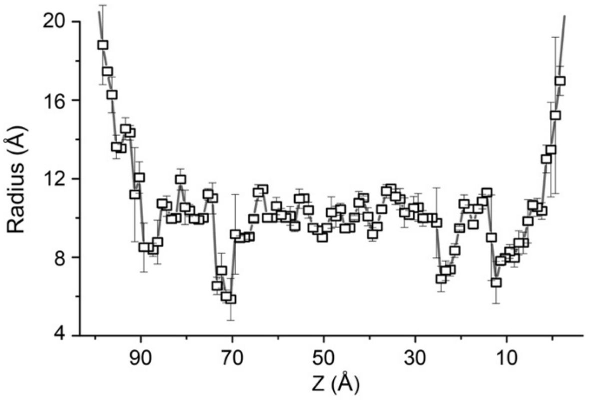

Supplementary Figure 8 | The average radial profile of the aerolysin channel. The average was taken over a 200 ns MD trajectory using the method described previously[4]. The error bars represent the standard deviations of the average radii due to the structure fluctuations of the channel.

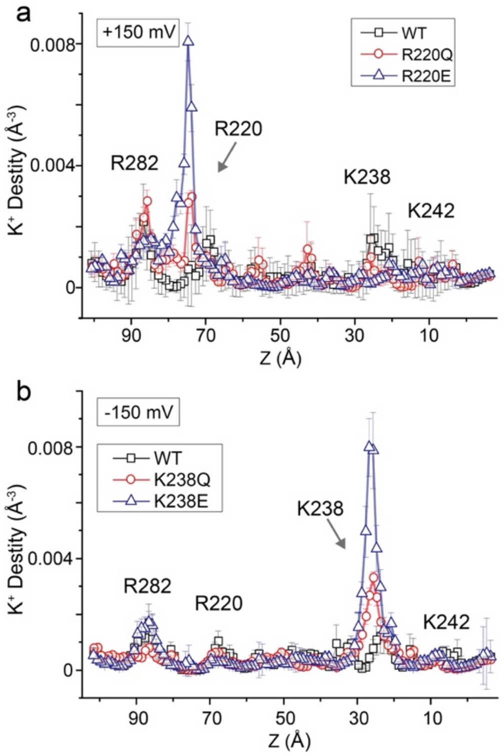

Supplementary Figure 9 | (a) The averaged density of K^+ in wt (black squares), R220Q (red circles), and R220E (blue triangles) at +150 mV along the z axis of the pore, as defined by the local radii profile shown in **Figure S8** during >200 ns MD simulation. (b) The density of K^+ in wt (black squares), K238Q (red circles), and K238E (blue tringles) at -150 mV.

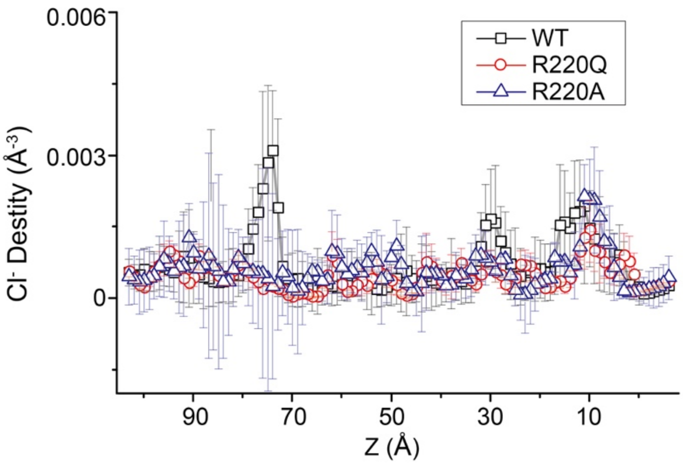

Supplementary Figure 10 | The averaged density of Cl^- in wt (black squares), R220Q (red circles), and R220A (blue triangles) at +150 mV along the z axis of the pore, as defined by the local radii profile shown in **Figure S8** during >200 ns MD simulation.

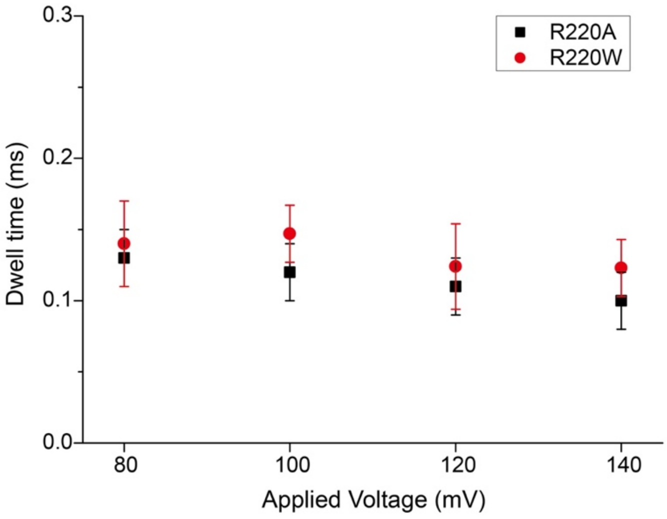

Supplementary Figure 11 | Dwell time of dA_4 translocating the R220A (a) and R220W (b) pores at different applied voltages.

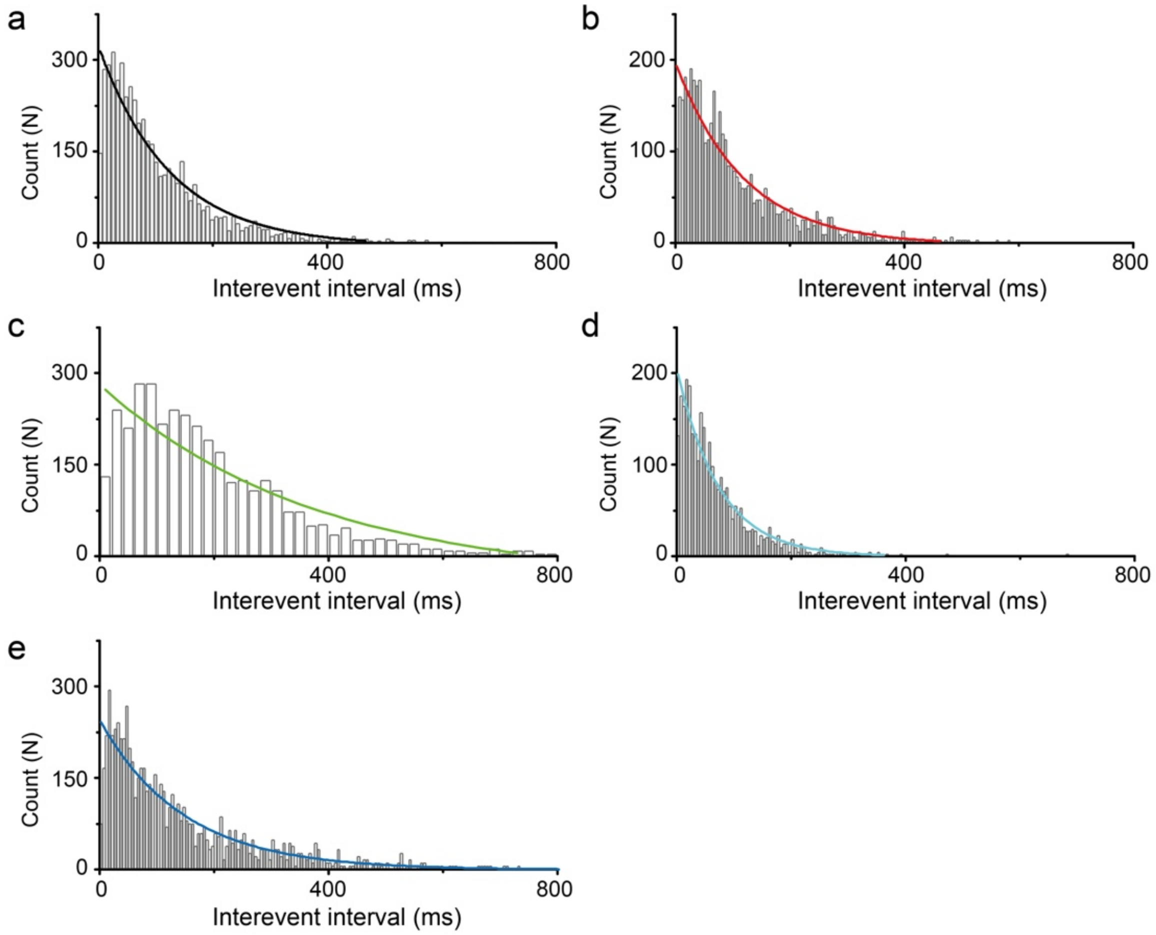

Supplementary Figure 12 | Inter-event interval (τ_{on}) distribution of dA_4 translocating across the (a) wt, (b) K238A, (c) K238Q, (d) K238N, and (e) K238R mutants. The fitted values are 125.1 ± 8.2 ms, 117.8 ± 5.4 ms, 373.5 ± 42.6 ms, 83.7 ± 2.0 ms, and 145.9 ± 6.9 ms from (a) to (e), respectively.

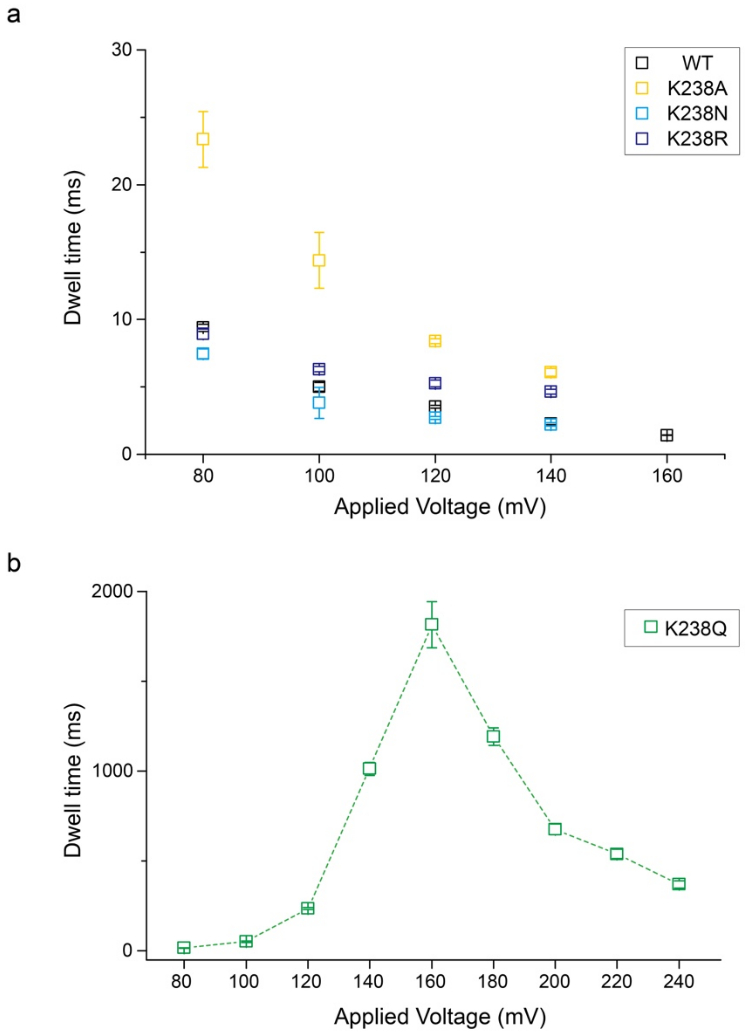

Supplementary Figure 13 | (a) Dwell time of dA_4 translocating the wt, K238A, K238N and K238R pores at different voltages. (b) Dwell time of dA_4 upon addition into the K238Q pore.

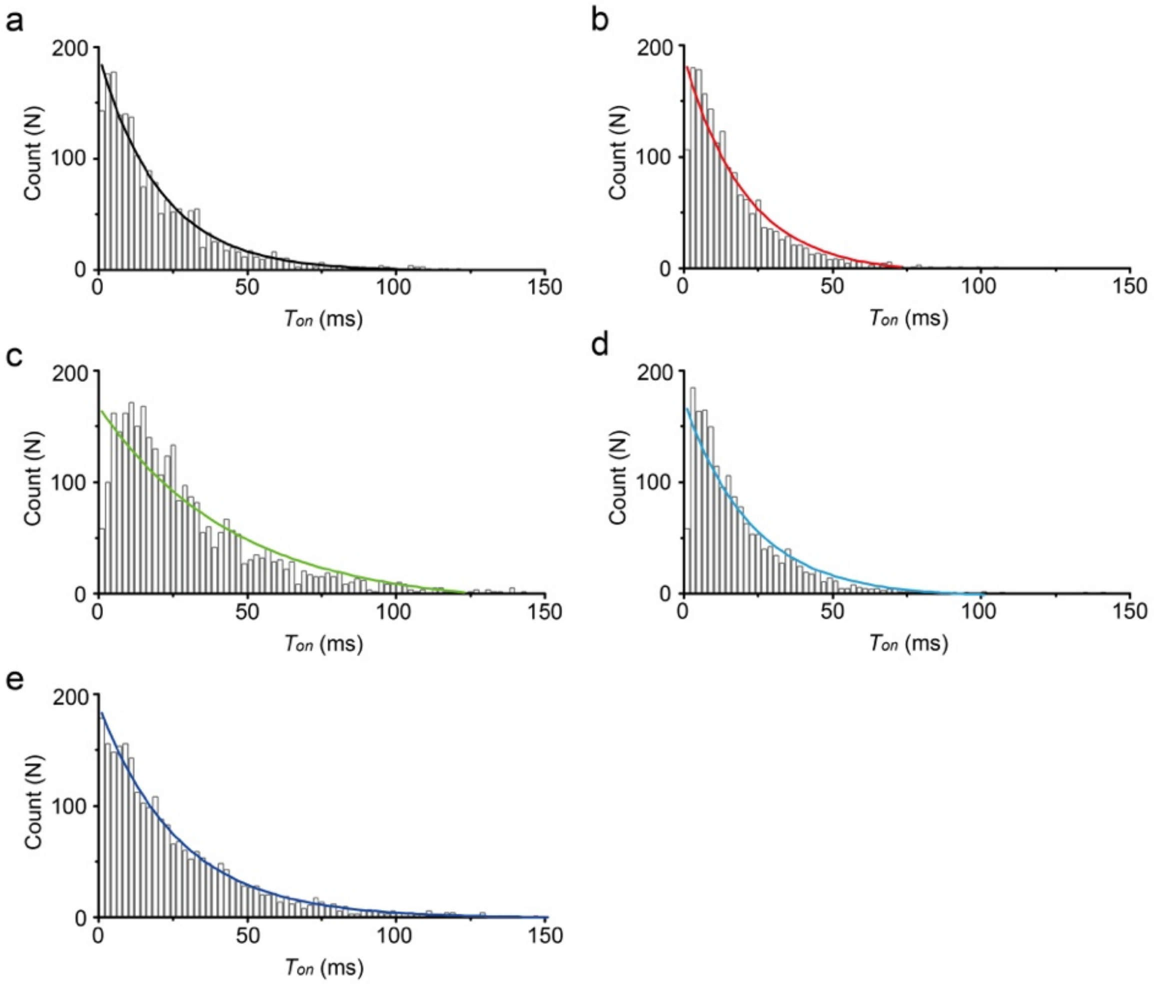

Supplementary Figure 14 | Inter-event interval (τ_{on}) distribution of EYQ3 peptide translocating across the (a) wt, (b) K238A, (c) K238Q, (d) K238N, and (e) K238R pore mutants. The fitted values are 20.8 ± 1.2 ms, 20.5 ± 1.6 ms, 45.3 ± 5.4 ms, 22.6 ± 2.2 ms, and 26.8 ± 0.6 ms from (a) to (e) , respectively.

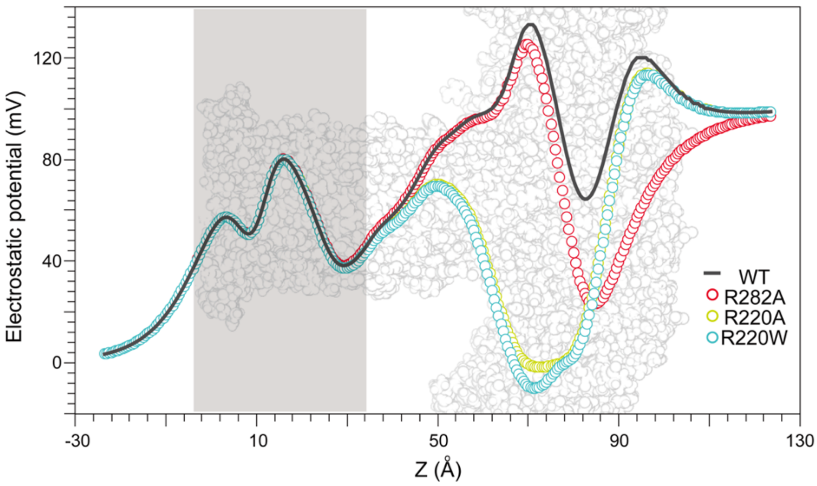

Supplementary Figure 15 | Electrostatic potential map along the pore lumen for WT, R282A, R220A, and R220W pores under an applied voltage of -100 mV. The membrane region is highlight by the gray background and the pore structure is indicated by the transparent surface representation.

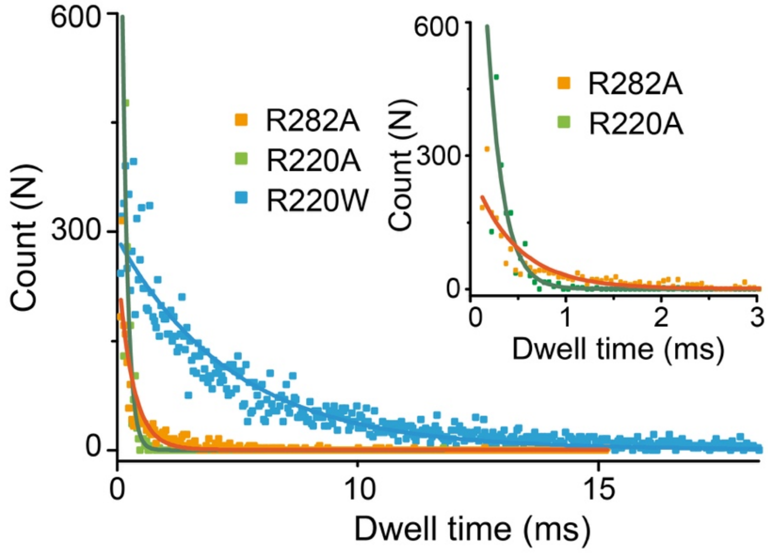

Supplementary Figure 16 | Dwell time distributions of HIV-1 Tat (47-57) through the R282A (black), R220A (red) and R220W (blue) mutant pores, respectively. The values were determined by the single exponential fitting.

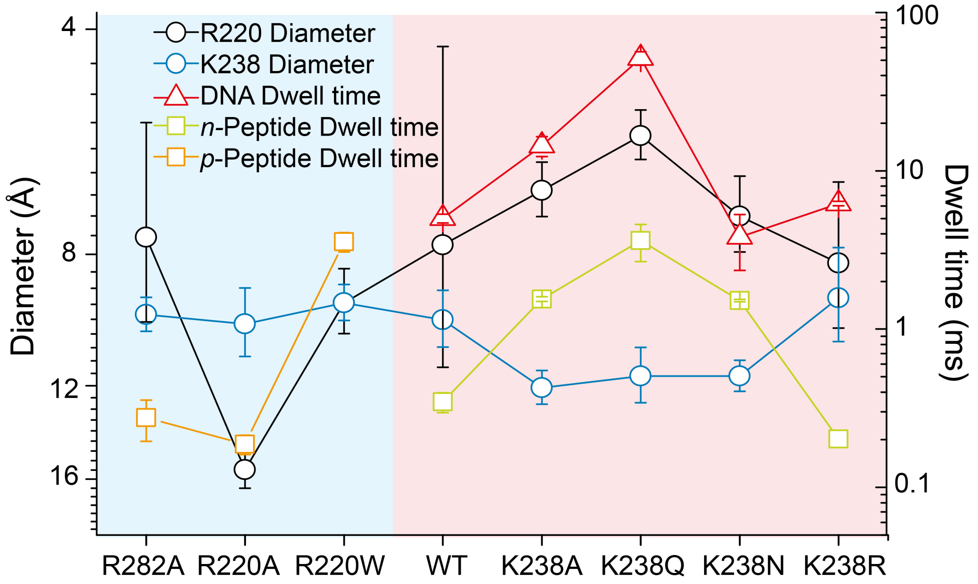

Supplementary Figure 17 | Summary comparison of diameter and dwell time of DNA and peptides (negatively and positively charged) transport through wt aerolysin and various mutants.

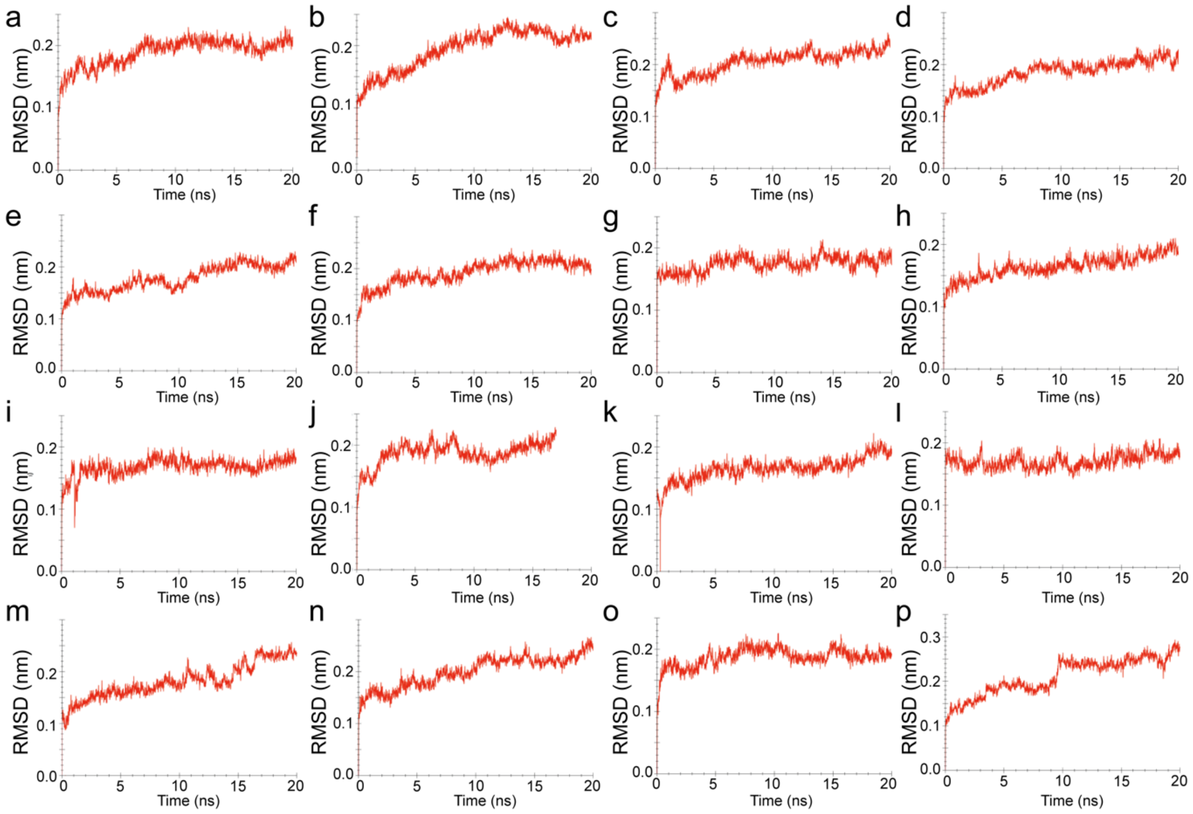

Supplementary Figure 18 | Root Mean Square Deviation (RMSD) of the backbone residues of the pore mutants along MD simulations. a) wt, b) R282A, c) R282W, d) R220A, e) R220E, f) R220K, g) R220Q, h) R220W, i) K238A, j) K238E, k) K238N, l) K238Q, m) K238R, n) K238W, o) K242A, p) K242W.

Supplementary References

1. Pellegrini-Calace, M., Maiwald, T. & Thornton, J.M. PoreWalker: A Novel Tool for the Identification and Characterization of Channels in Transmembrane Proteins from Their Three-Dimensional Structure. *PLOS Computational Biology* **5**, e1000440 (2009).
2. Cao C., Meng, Y.L., Cirauqui, N., Wang Y-Q., Dal Peraro, M., Tian H. & Long Y-T. Mapping the sensing spots of aerolysin for single oligonucleotides analysis. *Nature Communications* **9**, 2823 (2018).
3. Cirauqui, N., Abriata, L.A., van der Goot, F.G. & Dal Peraro, M. Structural, physicochemical and dynamic features conserved within the aerolysin pore-forming toxin family. *Scientific Reports* **7**, 13932 (2017).
4. Aksimentiev, A. & Schulten, K. Imaging α-Hemolysin with Molecular Dynamics: Ionic Conductance, Osmotic Permeability, and the Electrostatic Potential Map. *Biophysical Journal* **88**, 3745-3761 (2005).
